# The Spoils-system

**Published:** 1896-04

**Authors:** 


### THE LINGERING EMBERS OF THE SPOILS-SYSTEM.

If those Congressmen at Washington who have labored so hard to find some fault with and defame the good name of Secretary Morton would only point us to an administration of that department so economically and honestly administered, without any seed-scandals to explain, one could feel some tolerance for the miserable efforts to becloud the public mind and to rob the present administration of the courage and integrity Secretary Morton has exhibited in rooting out, tooth and nail, the infamous spoils-system that so long disgraced our whole governmental system, and of which the seed-scandal was only an outgrowth of this pernicious method of securing public place. If these public servants had half the zeal for our people and their own constituents that they endeavor to exhibit on our national parade-ground, the halls of Congress, they long years ago would have wiped away this wasteful and more than useless expenditure of public moneys that has brought shame and dishonor on our public servants, and exerted their influences to have this money used in providing greater safeguards for the agricultural and stock-raising industries by promptly establishing inspection of all horses sent abroad, and thus enlarge this market for a product whose price is not fixed in London for the whole world, and restore the values in our own country to a point approximating the cost of raising the stock.

